# Genetics of Microstructure of the Corpus Callosum in Older Adults

**DOI:** 10.1371/journal.pone.0113181

**Published:** 2014-12-16

**Authors:** Sri C. Kanchibhotla, Karen A. Mather, Anbupalam Thalamuthu, Lin Zhuang, Peter R. Schofield, John B. J. Kwok, David Ames, Margaret J. Wright, Julian N. Trollor, Wei Wen, Perminder S. Sachdev

**Affiliations:** 1 Centre for Healthy Brain Ageing, School of Psychiatry, University of New South Wales, Sydney, Australia; 2 Neuroscience Research Australia, Sydney, Australia; 3 School of Medical Science, University of New South Wales, Sydney, Australia; 4 National Ageing Research Institute, Royal Melbourne Hospital, Melbourne, Australia; 5 Academic Unit for Psychiatry of Old Age, University of Melbourne, Melbourne, Australia; 6 Queensland Institute Medical Research, Berghofer Medical Research Institute, Brisbane, Australia; 7 Department Developmental Disability Neuropsychiatry, University of New South Wales, Sydney, Australia; 8 Neuropsychiatric Institute, The Prince of Wales Hospital, Sydney, Australia; CSIRO, Australia

## Abstract

The current study sought to examine the relative influence of genetic and environmental factors on corpus callosum (CC) microstructure in a community sample of older adult twins. Analyses were undertaken in 284 healthy older twins (66% female; 79 MZ and 63 DZ pairs) from the Older Australian Twins Study. The average age of the sample was 69.82 (SD = 4.76) years. Brain imaging scans were collected and DTI measures were estimated for the whole CC as well as its five subregions. Parcellation of the CC was performed using Analyze. In addition, white matter lesion (WMLs) burden was estimated. Heritability and genetic correlation analyses were undertaken using the SOLAR software package. Age, sex, scanner, handedness and blood pressure were considered as covariates. Heritability (h^2^) analysis for the DTI metrics of whole CC, indicated significant h^2^ for fractional anisotropy (FA) (h^2^ = 0.56; p = 2.89×10^−10^), mean diffusivity (MD) (h^2^ = 0.52; p = 0.30×10^−6^), radial diffusivity (RD) (h^2^ = 0.49; p = 0.2×10^−6^) and axial diffusivity (AD) (h^2^ = 0.37; p = 8.15×10^−5^). We also performed bivariate genetic correlation analyses between (i) whole CC DTI measures and (ii) whole CC DTI measures with total brain WML burden. Across the DTI measures for the whole CC, MD and RD shared 84% of the common genetic variance, followed by MD- AD (77%), FA - RD (52%), RD - AD (37%) and FA – MD (11%). For total WMLs, significant genetic correlations indicated that there was 19% shared common genetic variance with whole CC MD, followed by CC RD (17%), CC AD (16%) and CC FA (5%). Our findings suggest that the CC microstructure is under moderate genetic control. There was also evidence of shared genetic factors between the CC DTI measures. In contrast, there was less shared genetic variance between WMLs and the CC DTI metrics, suggesting fewer common genetic variants.

## Introduction

The corpus callosum (CC) is the largest white matter (WM) tract connecting the two cerebral hemispheres and contains more than 2×10^8^ axons [1], [2]. The size and myelination of these fibres determine the time taken for inter-hemispheric transfer of information [3].

Diffusion tensor imaging (DTI) is used to study WM integrity, and it provides quantitative three-dimensional analyses of WM microstructure [4], [5]. Different DTI measures such as anisotropy (fractional anisotropy-FA) and diffusivity (mean diffusivity- MD; radial diffusivity- RD and axial diffusivity- AD) can be obtained, each of which is sensitive to different aspects of WM integrity, including levels of myelination (FA, RD), axonal density/diameter (FA), axonal damage or loss (AD) [6]–[10].

Ageing leads to macro and microstructural changes to fibres in the CC, affecting inter-hemispheric processing [11]. Although the number of fibres in the CC does not change from birth [12], their size, density [2] and composition (myelination) [12] varies with age [1]. For the CC, greater atrophy using DTI measures has been observed with increasing age in the anterior and mid-body regions compared to posterior regions [5], [13], [14].

Age-related changes in the CC [15], [16] have been associated with age-related cognitive impairment [17]–[19], reduced processing speed [20], bimanual motor decline [21] and neurodegenerative disease [22]–[25]. Moreover, the study of WM integrity measures in older individuals may help in the early diagnosis of disease such as Alzheimer's disease and mild cognitive impairment [26]–[28] and may serve as biomarkers to differentiate them at an early stage [29]. Hence, it is important to gain a better understanding of the role of genetic and environmental factors in age-related integrity of the CC. Further, studying the genetics of CC in older individuals may help to understand the aetiology of the age-related degeneration of CC and also clarify the relationship between its microstructure, function and disease.

Heritability studies provide evidence for the role of genes in WM integrity [30]. To date, however, the heritability of CC in older individuals has been reported in only two studies, one examining FA in older males only [31], while the other used an extended family study design [32]. In a small sample of older males (n = 64), the heritability for FA of the CC splenium (67%) was found to be more than that of the genu (49%) [31]. However, in an extended family study (n = 467), which included older adults (age range 19–85 yrs), heritability of FA across the lifespan for the genu was high (66%), with heritability values of FA for the body of CC and splenium ranging from 54–57%. Also, CC RD was reported to be heritable (37%), but not AD [32]. The heritability of MD was not reported in either of these two studies. Therefore, more studies are required to examine heritability of all CC DTI measures using larger samples of older adults and including females.

One major consideration in the study of the CC is how best to divide it into sub regions (parcellation) it. Many of the previous reports only studied the whole CC as a single unit or divided it into two (genu & splenium) or three (genu, mid body& splenium) regions. However, the CC has no clear anatomical boundaries [1], and it varies highly in size and shape among individuals [12], [33]–[35]. The widely accepted Witelson's classification [36], and other geometric parcellation methods [37], [38], are not based on fibre composition and connections and may therefore not exactly reflect the functional regions of CC [39]. Although there are several other methods of parcellation [40]–[42], these methods are based on their connections to the target regions in the cortex, leading to inconsistent sub divisions of CC. Chao et al., [39] proposed parcellation of the CC based on high angular resolution diffusion imaging (HARDI) and the neural connections of CC with distinct cerebral areas, sub divided further based on differences in cell layers and structures. When using this method, the CC is parcellated based on its connections to distinct brain functional units. An added advantage is that HARDI can assist in resolving even lateral and crossing fibres. Hence, this probabilistic topographical method helps to better understand the fibre composition of the CC [39].

WM lesions (WMLs) are a marker of WM pathology and are commonly observed as hyperintense regions in T2-weighted or fluid attenuated inversion recovery (FLAIR) scans in older adults. WMLs are thought to reflect small vessel disease but their aetiology is incompletely understood [43], [44]. WMLs have moderate to high heritability and are influenced by both genetic and environmental factors [45]. Although, several studies reported an association of the development of these WMLs with WM integrity measures across several regions of the brain [46]–[48], it is unclear whether common genetic factors influence WMLs and age-related WM integrity measures. To date, there are no studies specifically reporting the phenotypic or genetic correlations between CC DTI metrics and WMLs.

In this study, we estimate the relative influence of genetic and environmental factors on DTI measures for the entire CC and for five parcellated regions using the method of Chao et al., [39] in a community-dwelling sample of older adult twins. Examination of five regions of the CC based on anatomical and cortical connections will enable a hitherto more detailed examination of heritability than previous studies. Additionally, in contrast to the study of Pfefferbaum et al., [31] our sample contains both women and men aged 65 and above. In an extension of this, we aimed to determine whether there are common genetic influences across various DTI measures for whole CC and with WMLs, a marker of WM atrophy.

## Methods

### A. Ethics statement

The study was approved by the Australian Twin Registry, University of New South Wales, University of Melbourne, Queensland Institute of Medical Research and the South Eastern Sydney and Illawarra Area Health Service and written informed consent provided by all participants. Participant's information was de-identified and anonymized prior to analysis.

### B. Participants

The current study is comprised of 142 twin pairs (MZ male = 29 pairs; MZ female = 50 pairs; DZ male = 8 pairs; DZ female  = 33 pairs, DZ mixed  = 22 pairs) drawn from the longitudinal Older Australian Twin Study (OATS) with available brain imaging data. The mean age of the sample used in this study was 69.82 (+/− 4.76) years with ages ranging from 65 to 85. All participants were Caucasian. In the current analysis, individuals with a consensus diagnosis of dementia were also excluded.

Briefly, OATS recruited twins aged 65 years and above who were registered with the Australian Twin Registry and through recruitment drives across the three eastern states of Australia – New South Wales, Queensland and Victoria. Other inclusion criteria were ability to complete questionnaires in English and having a consenting co-twin. Individuals were excluded if they were diagnosed with any progressive malignancy or other life threatening illness or acute psychiatric disorder. The study was approved by the relevant ethics committees and written informed consent provided by all participants. The full details of the study have been published elsewhere [49]. The zygosity of the sample was assessed based on identity by descent using available genome-wide genotyping data along with self-report data [50].

Participants completed an extensive face-to-face interview, which included demographic, medical and health information. Data on alcohol consumption, tobacco smoking and diabetes were obtained by self-report questionnaires [49]. Fasting peripheral blood samples were obtained and assessed for total cholesterol (using a Beckman LX20 Analyser) and glucose levels (glucose oxidase method, Beckman Coulter, Fullerton, CA). Individuals whose fasting blood glucose level was ≥7 mmol/L or had been diagnosed by a medical practitioner as diabetic and were currently on a diabetic diet, hypoglycaemic tablets or insulin were categorised as diabetic. Two seated readings of systolic and diastolic blood pressure (BP) were obtained by the interviewer and averaged. For current smoking status, individuals were classified as either a current smoker or not. Daily alcohol consumption was based on the frequency of drinking and number of drinks (10 g alcohol) consumed on typical drinking days. Participants were classified into two categories: (i) ≤1 drink/day; (ii)>1 drink/day. For handedness, individuals were categorised either as right-handed or not, based on a series of questions regarding which hand they used for various daily activities. As there were very few left handed and mixed individuals in the sample, we combined them as non-right handed for our analysis (N = 18).

### C. Brain MRI Scanning

MRI data were obtained on three 1.5 Tesla scanners and a 3 Tesla scanner owing to the multi-site nature of this study. Co-twins were imaged on the same scanner. We used Siemens Magnetom Avanto and Sonata scanners (Siemens Medical Solutions, Malvern PA, USA) with similar years of manufacture and upgrade in centres 2 (Victoria - 97 participants) and 3 (Queensland- 79 participants), respectively. In centre 1 (New South Wales), we initially used a 1.5 T Philips Gyroscan scanner (Philips Medical Systems, Best, Netherlands) (83 participants) and subsequently a 3 Tesla Philips Achieva Quasar Dual scanner (25 participants). The acquisition protocols and parameters were tested and matched between the centres through standardization of spatial resolution and slice thickness, using a 3D phantom to correct geometric distortions and using five volunteers who were scanned on the four scanners [49]. Twin pairs were scanned on the same day or temporally very close to each other (<few weeks).

Diffusion weighted MRIs (DWI) scans were used for computing the diffusion tensor imaging (DTI) measures. DWI sequences were performed using a similar protocol for the 1.5 Tesla scanners in the three centres. To increase the signal-to-noise ratio (SNR), all of the subjects were scanned twice for DWI sequences in the same MRI session. A single-shot echo-planar imaging (EPI) sequence (TR = 12729 ms, TE = 76 ms) was used. Diffusion sensitizing gradients were applied along 32 non-collinear directions (b1 = 800 s/mm^2^), together with a non-diffusion-weighted acquisition b0. For each DWI scan, 55 axial slices were collected. The field of view was 240 mmx240 mmx137.5 mm with acquisition matrix 96×96, and slice thickness 2.5 mm with no gap between the slices, yielding 2.5 mm^3^ voxels of isotropic sizes. Two extra non-diffusion-weighted (b0) EPIs were separately acquired and then combined with DWI scans for higher SNR. TR = 7115 ms and TE = 70 ms and b1 = 1000 s/mm^2^ were used for the 3 Tesla Philips Achieva Quasar Dual scanner at Centre 1 while the other DWI parameters were the same.

We used T2-weighted fluid attenuated inversion recovery (FLAIR) and 3D T1-weighted MRIs scans for the computation of white matter lesion (WMLs) volumes. 3D T1-weighted volumetric sequence was performed using a similar protocol in the three centres with in-plane resolution = 1×1 mm, slice thickness = 1.5 mm, slice number = 144, TR = 1530 ms, TE = 3.24 ms, TI (Inversion time) = 780 ms, and flip angle = 8. Two T1-3D scans were acquired for each participant for an increased SNR. FLAIR scans main acquisition parameters were: TR/TE = 10000/120 ms, inversion delay = 2500 ms, in-plane resolution 0.898×0.899 mm^2^, slice thickness = 3.0 mm, with 50 axial slices.

### D. DTI Processing

The DTI data were pre-processed using FMRIB's Software Library (FSL) version 5.0 [51], [52], which included three main steps. Firstly, eddy current correction, which includes correction of DTI images due to head distortions and for different gradient directions; secondly, brain extraction, which includes the deletion of non- brain tissue, using BET (Brain Extraction Tool); and thirdly, fitting tensors using the DTIfit program, which includes the process of fitting the diffusion tensor model across each voxel of the pre-processed DTI data to derive anisotropic (FA) and diffusivity (MD, RD and AD) images. These images were then checked individually for any artefacts or missing slices. Any samples that failed quality control checks were removed from the analysis.

### E. Tracing and parcellation of corpus callosum

**Figure 1:**
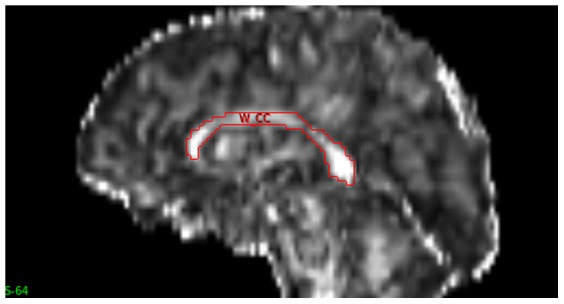
Mid- sagittal slice showing tracing of the whole corpus callosum.

**Figure 2:**
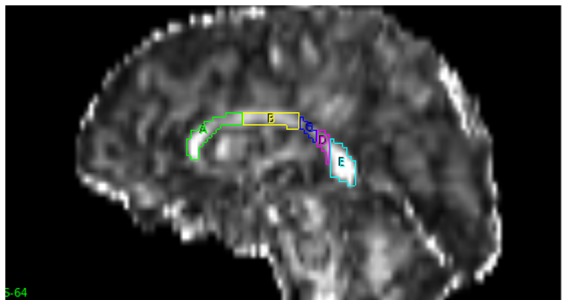
Mid- sagittal slice showing the divisions of corpus callosum into 5 regions anterior to posterior (A–E) using the parcellation method of Chao et al. [39].

A semi-automated method of tracing and parcellation of the CC was performed using Analyze version 11.0 (http://www.analyzedirect.com/) by a trained researcher who was blinded to family relationship. Initially, the pre-processed DTI images were adjusted to note the mid-sagittal slice number using volume render and fly tools. The auto-trace tool was used to trace whole CC in the mid-sagittal slice of FA images (Fig. 1). The traced CC was then parcelled into five vertical partitions (A–E) from anterior to posterior, as proposed by Chao et al., [39] using the grid divider tool (Fig. 2): A - frontal regions; B - pre motor & supplementary motor; C - motor; D - sensory; and E - parietal, temporal & occipital. The same object maps were loaded on MD, RD and AD images to obtain the respective DTI measures.

Tracing and parcellation were done individually on both images (i.e. scan 1 and 2 for each participant). The two sets of data were significantly correlated (r>0.8, p<0.05). The four DTI metrics were calculated using the three eigen vectors extracted from the DTI images. The mathematical formulae are shown in S1 File and show that all of the metrics are related and not independent. FA and MD values are derived from all three eigen vectors (λ_1_, λ_2_, λ_3_), whilst RD is calculated from the average of two eigen vectors (λ_2_ and λ_3_) whilst λ_1_ is taken as AD. The two sets of data (scans 1 & 2) were highly correlated (r≥0.85). Here, we report the heritability results based on the averages of the two scans. Additional analyses of the individual scans are presented in S1 File.

### F. Measurement of WMLs

WMLs were delineated from FLAIR and 3D T1-weighted structural scans using an automated pipeline described in detail previously [44], [53]. Briefly, the automatic quantification of WMLs was carried out in the following steps: 1) co-registration of each participant's FLAIR images to their corresponding T1-weighted structural images; 2) segmentation of T1-weigthed structural images into three separate tissue components, i.e. grey matter, white matter and cerebrospinal fluid; 3) removal of non-brain tissue from both T1-weighted and co-registered FLAIR images using a brain mask (computer generated, which is used to discriminate between brain from non-brain tissue) transformed from the average mask originally defined in the Talairach space (a 3-dimensional atlas of the brain); 4) inverting the spatial normalization transformation to produce the brain masks and WM probability maps in the individual imaging space for the WMLs detection and non-brain tissue removal; 5) intensity correction of both FLAIR and T1-weighted images after the removal of non-brain tissues and then 6) segmentation of WMLs using FLAIR scans.

### G. Statistical analysis

The twin method was used to study the influence of genetic and environmental factors on a particular trait. Monozygotic (MZ) twins share all genes whereas dizygotic (DZ) twins share only half of their genes. By comparing the similarity between the identical and non-identical twin pairs for a particular trait, the effect of genetic, shared and non-shared factors can be estimated. We calculated heritability of the four DTI measures (FA, MD, RD and AD) across whole CC and its five sub regions as described below.

#### Covariates

Age and scanner were significantly associated with the four DTI measures of whole CC (p≤0.05). Sex was significantly correlated with MD, AD and RD of whole CC (p≤0.05), but not FA (p>0.05). Handedness was significantly associated with MD and RD of whole CC (p≤0.05) but not with whole CC FA (p>0.05). Previous studies have identified risk factors for the integrity of WM across various regions of brain, which include hypertension, serum cholesterol [54]–[56], alcohol consumption [57], tobacco smoking [58] and diabetes [59], [60]. Of these potential confounders, only diastolic and systolic BP were significantly correlated with FA of the whole CC (p≤0.05). The other risk factors - total cholesterol, diabetes, alcohol consumption and smoking status - were not significantly associated with any of the DTI measures (p>0.05). For the region-wise analysis of the CC sub regions, there was no specific pattern of association (see S1a Table and S1b Table in S1 File). To be consistent across analyses the following were used as covariates: age, sex, scanner, handedness, diastolic and systolic BP.

#### Heritability and genetic correlations

To estimate heritability and genetic correlations, the SOLAR (Sequential Oligogenic Linkage Analysis Routines) software package was used, which is based on the variance components method (http://solar.txbiomedgenetics.org/) [61]. The variance components model used here for heritability estimation includes only the genetic and environmental variance components. The shared environmental variance can be measured by adding an additional component of variance to the model. However, under the twin design, a very large sample is required to distinguish between those two models [62]. Since the shared environment component was not significant in our sample in other neuroimaging analyses, we therefore have used only the genetic and environment components for consistency [similar to 63]. Multiple traits can be analysed by extending the variance component model [64]–[66]. Heritability was also estimated separately for women (N = 166) but not for men due to the relatively small sample size (N = 74) and the imbalance between the number of MZ and DZ pairs (∼4∶1).

Bivariate genetic correlations were assessed between (i) whole CC DTI metrics and (ii) whole CC DTI measures and total WML burden. Analyses were undertaken controlling for the covariates listed above.

## Results

The demographic details of the sample are shown in Table 1. There were more females (66.19%) than males. There was no significant difference between MZ and DZ pairs for all relevant variables (p>0.5) including age (p = 0.79), sex (p = 0.82) and handedness (p = 0.88). The entire sample was mainly right-handed (89.75%), nearly one-third of the sample were current smokers (29.07%) and less than 14% were diabetic. The descriptive statistics of the DTI measures for the whole CC and its sub- regions are shown in Table 2. Total WMLs were significantly correlated with all the DTI metrics across the whole CC.

**Table 1:** Demographics and Characteristics of the Twin Sample.

	Total sample	MZ	DZ	p-values
N	284	158	126	
Age M (SD) yrs	69.82 (4.76)	69.92 (4.97)	69.69 (4.51)	0.79
Age Min/Max yrs	65/85	65/83	65/85	-
Sex - Female, N	188	100	88	0.39
Right-handed, N	254	140	114	0.87
Current smokers, N	82	50	32	0.40
Diabetic, N	32	21	11	0.23
Alcohol consumption>1 drink per day, N	66	43	23	0.20
Cholesterol (mmol/L) mean (SD)	5.20 (1.05)	5.10 (1.04)	5.33 (1.06)	0.58
Systolic mean (SD)	138.17 (18.15)	137.84 (18.92)	138.57 (17.21)	0.79
Diastolic mean (SD)	80.77 (10.35)	79.77 (9.95)	82.00 (10.75)	0.14
Total white matter lesions (mm^3^)	6688.04 (7997.89)	6513.22 (7977.05)	6907.26 (8050.41)	0.12

Notes: MZ – monozygotic twins; DZ- Dizygotic twins; M –mean; SD – standard deviation.

**Table 2:** Descriptive Statistics for the DTI measures.

	FA		MD (mm^2^/s)		RD (mm^2^/s)		AD (mm^2^/s)	
CC Region	Mean	SD	Mean	SD	Mean	SD	Mean	SD
Whole CC	5.42×10^−1^	4.8×10 ^−2^	1.06×10 ^−3^	1.3×10 ^−4^	0.73×10 ^−3^	1.1×10 ^−4^	0.31×10 ^−2^	7.5×10 ^−4^
A^a^	5.13×10^−1^	5.1 ×10 ^−2^	1.08×10 ^−3^	1.5×10 ^−4^	0.77×10 ^−3^	1.3×10 ^−4^	0.30×10 ^−2^	7.6×10 ^−4^
B^a^	5.07×10^−1^	5.6×10 ^−2^	1.07×10 ^−3^	1.4×10 ^−4^	0.77×10 ^−3^	1.3×10 ^−4^	0.31×10 ^−2^	8.0×10 ^−4^
C^a^	4.63×10^−1^	7.2×10 ^−2^	1.18×10 ^−3^	1.7×10 ^−4^	0.88×10 ^−3^	1.7×10 ^−4^	0.32×10 ^−2^	7.9×10 ^−4^
D^a^	5.27×10^−1^	7.3×10 ^−2^	1.12×10 ^−3^	1.6×10 ^−4^	0.78×10 ^−3^	1.6×10 ^−4^	0.32×10 ^−2^	8.1×10 ^−4^
E^a^	6.21×10^−1^	5.7×10 ^−2^	0.99×10 ^−3^	1.4×10 ^−4^	0.62×10 ^−3^	1.1×10 ^−4^	0.31×10 ^−2^	8.0×10 ^−4^

Notes: FA – Fractional anisotropy; MD- Mean diffusivity; RD- Radial diffusivity; AD – Axial diffusivity; SD – standard deviation; CC – corpus callosum. The means are calculated considering all the voxels within that subregion^a^.

### A. Heritability analysis

Intra-class correlations (ICC) estimated for MZ and DZ twin groups (95% CI) for whole CC DTI measures are shown in Table 3. Since data was acquired and averaged from two separate scans of each individual, ICCs were also calculated for the major measures for the whole CC for comparison for each scan (see S2a Table and S2b Table in S1 File). Although the values for each scan differ slightly, the estimates and resulting heritability values were similar (see S3a Table and S3b Table in S1 File). The heritability estimates of the four DTI measures for whole CC and its five sub regions from anterior to posterior (A–E), are shown in Table 4. For whole CC, moderate heritability was observed for all the four measures, FA, MD, RD and AD (h^2^ = 0.37–0.56). For the region-wise analysis, similar to the entire CC, FA, MD and RD are heritable. However, AD is heritable only in the anterior CC (region A).

**Table 3:** Intra-class correlations (ICC) estimates for MZ and DZ twin groups for whole CC DTI measures.

Whole CC	MZ ICC (95% C.I.)	DZ ICC (95% C.I.)
FA	0.62 (0.46–0.74)	0.39 (0.15–0.58)
MD	0.75 (0.63–0.83)	0.63 (0.45–0.76)
RD	0.65 (0.50–0.76)	0.46 (0.24–0.63)
AD	0.67 (0.53–0.78)	0.73 (0.59–0.83)

Notes: MZ – monozygotic twins; DZ- Dizygotic twins; FA – Fractional anisotropy; MD- Mean diffusivity; RD- radial diffusivity; AD – Axial diffusivity; ICC- Intra-class correlations.

**Table 4:** Heritability estimates for Whole CC and its five sub-regions [A-E] for the four DTI measures.

CC Region	FA		MD		RD		AD	
	h^2^±SE	p-value	h^2^±SE	p-value	h^2^±SE	p-value	h^2^±SE	p-value
Whole CC	0.56±0.07	2.89×10^−10^ *	0.52±0.08	0.30×10^−6^ *	0.49±0.08	0.20×10^−6^ *	0.37±0.09	8.15×10^−5^ *
A	0.54±0.07	2.52×10^−9^ *	0.46±0.09	1.57×10^−5^ *	0.44±0.09	1.56×10^−5^ *	0.26±0.10	0.08×10^−1^
B	0.44±0.08	0.90×10^−−6^ *	0.44±0.09	2.39×10^−5^ *	0.48±0.09	0.50×10^−6^ *	0.15±0.09	0.05
C	0.34±0.08	2.18×10^−4^ *	0.36±0.09	1.38×10^−4^ *	0.39±0.08	2.19×10^−5^ *	0.07±0.10	0.24
D	0.32±0.08	2.27×10^−4^ *	0.35±0.08	0.85×10^−4^ *	0.38±0.08	1.81×10^−5^ *	0.10±0.10	0.16
E	0.49±0.07	4.15×10^−8^ *	0.40±0.09	2.65×10^−5^ *	0.40±0.08	5.30×10^−6^ *	0.15±0.10	0.07

*Values are significant at p<.0001.

Notes: FA – Fractional anisotropy; MD- Mean diffusivity; RD- radial diffusivity; AD – Axial diffusivity; h^2^- heritability estimate; SE – standard error.

Significant heritability for women was observed for all four DTI measures ranging from 0.39–0.68 for the whole CC (see S4 Table in S1 File, N = 50 female MZ pairs and 33 DZ pairs).

### B. Genetic correlation analysis

Bivariate genetic correlation analysis was performed for the whole CC between the four DTI measures [Table 5] and separately for each scan (see S5a Table and S5b Table in S1 File). In general, significant genetic correlations were observed between FA, MD, RD and AD. However, AD did not show a significant correlation with FA. FA shared around 11% (ρ_g_ = −0.34) and 51% (ρ_g_ = −0.72) common genetic variance with MD and RD respectively. The negative sign of the correlation coefficient indicates opposing effects of the shared genetic factors on the measures, i.e. there are some shared genetic factors that are associated with an increase in FA and a decrease in MD or RD [32]. MD shared 85% of common genetic variance (ρ_g_ = 0.92) with RD and 77% of common genetic variance (ρ_g_ = 0.88) with AD in the same direction. Similarly RD and AD shared 37% common genetic variance (ρ_g_ = 0.61).

**Table 5:** Genetic (upper matrix) and environmental correlations (lower matrix) of whole CC DTI measures and total WMLs.

	FA (ρ_g_±SE) [p]	MD (ρ_g_±SE) [p]	RD (ρ_g_±SE) [p]	AD (ρ_g_±SE) [p]	WMLs (ρ_g_±SE) [p]
**FA (ρ_e_**±**SE) [p]**	1	−0.34±0.11 [0.01]*	−0.72±0.07 [2.74×10^−6^]*	0.08±0.15 [0.58]	−0.23±0.11 [0.04]
**MD (ρ_e_**±**SE) [p]**	−0.61±0.07 [9.78×10^−10^]*	1	0.92±0.02 [1.15×10^−6^]*	0.88±0.07 [3.17 ×10^−6^]*	0.44±0.13 [0.6×10^−3^]*
**RD (ρ_e_**±**SE) [p]**	−0.79±0.04 [2.01×10^−22^]*	0.90±0.02 [2.01×10^−53^]*	1	0.61±0.12 [0.31 ×10^−3^]*	0.42±0.13 [0.89×10^−3^]*
**AD (ρ_e_**±**SE) [p]**	0.10±0.10 [0.29]	0.52±0.07 [1.67×10^−9^]*	0.24±0.09 [0.01]	1	0.40±0.13 [0.32×10^−2^]*
**WMLs (ρ_e_**±**SE) [p]**	0.03±0.11 [0.79]	−0.13±0.11 [0.26]	−0.109±0.11 [0.39]	0.03±0.10 [0.76]	1

*Values are significant at p<0.01.

Notes: FA – Fractional anisotropy; MD- Mean diffusivity; RD- radial diffusivity; AD – Axial diffusivity; WMLs – White matter lesions; ρ_g_ – genetic correlation coefficient; ρ_e_ - environmental correlations; SE – standard error.

High heritability was observed for total brain WMLs (h^2^ = 0.73, SE = 0.051, p = 3.03×10^−15^). Bivariate genetic correlation analysis of the four DTI measures of the whole CC with total brain WMLs showed significant correlations for WMLs with all four DTI CC measures [Table 5]. Total brain WMLs shared 5% common genetic variance with FA with an inverse correlation observed (ρ_g_ = −0.23), indicating an opposing effect. On the other hand, positive genetic correlations were also observed with total brain WMLs sharing 19% common genetic variance with MD (ρ_g_ = 0.44), 17% with RD (ρ_g_ = 0.42) and 16% with AD (ρ_g_ = 0.40).

## Discussion

Our study of community-dwelling older adult twins aimed to estimate the role of genetic factors on the microstructure of the CC and its five sub regions using DTI. In addition, we estimated the genetic correlations between whole CC DTI measures and with WMLs.

The heritability analysis indicated that all four DTI measures (FA, MD, RD and AD) for whole CC are moderately heritable. Our results are in agreement with the results of previous studies of older adults where FA and RD were heritable. In general, we also observed AD to be heritable in older individuals, which is contrary to the study by Kochunov et al. [32]. Differences between the two studies may explain the discrepancy in results. Here, we examined older twins only, whilst Kochunov et al. examined CC across the lifespan in a family study. Moreover, these prior studies did not examine the heritability of MD; we found MD to have moderate heritability [31], [32].

Our results vary from a previous study [31], which reported higher genetic contribution for the FA in the posterior CC (splenium h^2^- 67%) than the anterior CC (genu h^2^ -49%). We did not observe such high heritability in the posterior regions of the CC. This may be due to differences in methods of tracing and parcellation, and the fact our sample is slightly younger (mean age of our sample 69.8 years vs 76 years) and includes both sexes. However, our results are similar to the results of Kochunov et al., [32], which used an extended family sample.

Genetic factors account for approximately 56% of the variance in CC FA, which may be sensitive to levels of myelination, axonal density and its diameter [6]–[10]. Similarly for MD, RD and AD, genetic factors contribute about 50% of the observed variability. Likewise, region wise analyses for the heritability of DTI measures across CC also indicated that heritability values for FA, MD and RD showed moderate heritability (31–54%). Moderate heritability was observed for AD but only in the anterior CC (region A).

We studied bivariate genetic correlation analysis between the DTI metrics for the whole CC. In our analysis, MD-RD shared the highest common genetic variance (84%), followed by MD- AD (77%), FA - RD (52%), RD - AD (37%) and FA – MD (11%). The relationship between MD and RD may be explained by the strong mathematical relationship between these two measures. In the DTI analysis, the magnitude and direction of maximum and minimum water diffusion at each voxel are quantified as three Eigen values at each voxel. MD being the average of the three Eigen values provides the measure of rate of water diffusion whereas RD, which is the average of the second and third Eigen values, is a measure of transverse diffusion. AD is the largest of the three Eigen values and is a measure of the rate of diffusion along the primary axis. As discussed previously, different DTI measures may be sensitive to different aspects of WM integrity and a better understanding of the relationships between these DTI measures may help to understand the underlying changes occurring in axons and myelin in older individuals. However, the relationship between the DTI measures and the tissue microstructural properties are not yet completely understood [67], [68].

Bivariate genetic correlation analysis of total brain WMLs with whole CC DTI metrics also indicated some common genetic factors between them, with the highest shared common genetic variance observed with MD and RD. The significant genetic correlations observed may reflect shared genetic variance that contributes to common pathological processes leading to age-related WM pathology. WMLs are usually regarded as markers of small vessel disease [69] although their pathology is not completely understood [45]. However, the CC is less affected by small vessel disease [70]. Therefore, the common genetic factors between these measures, as reported in our results, may possibly indicate some other genetic factors for WMLs that are independent of small vessel disease.

This study has a few limitations. Firstly, the DTI metrics were for CC only, while the measures of WMLs, which are not generally observed in CC, were for the total brain. Another limitation is that the MRI scans were collected across three states and used four scanners. However, this is a relatively common issue in multicenter DTI studies and we scanned the twin pairs on the same scanner and also adjusted for the scanner in our analyses. Secondly, we did not explore the shared genetic variance between WM integrity of the CC and its connected cortical regions, as measured by cortical thickness, which may assist in the clarification of the genetic basis of the connectivity of CC. Thirdly, we performed a cross-sectional study. It would be of interest to examine the heritability of longitudinal change in the integrity of CC. And lastly, we note that the “environment” component calculated in heritability estimates not only includes environmental variation but also any measurement error in the phenotypes measured.

In conclusion, we observed that in general, the CC microstructure as measured by DTI is under moderate genetic control in older adults. All the CC DTI metrics FA, MD, RD and AD showed significant heritability for whole CC. These measures may serve as useful phenotypes to identify specific genetic variants associated with CC integrity in older individuals. Additionally, we observed some common genetic factors between whole CC DTI metrics and also with CC DTI measures and WMLs, which may lead to further investigations to uncover common genes contributing to age-related changes in WM microstructure in the future.

## Supporting Information

S1 File **This file contains S1 Table–S5 Table.** S1a Table, Summary of covariate results used in the variance components model for whole CC and its sub regions (FA & MD). S1b Table, Summary of covariate results used in the variance components model for whole CC and its sub regions (RD & AD). S2a Table, Intra-class correlations (ICC) estimates for MZ and DZ twin groups for whole CC DTI measures for Scan 1. S2b Table, Intra-class correlations (ICC) estimates for MZ and DZ twin groups for whole CC DTI measures for Scan 2. S3a Table, Heritability estimates for Whole CC and its five sub-regions [A–E] for the four DTI measures for scan 1. S3b Table, Heritability estimates for Whole CC and its five sub-regions [A–E] for the four DTI measures for scan 2. S4 Table, Heritability estimates for the four DTI measures of whole CC in females. S5a Table, Genetic (upper matrix) and environmental correlations (lower matrix) of whole CC DTI measures and total WMLs for scan 1. S5b Table, Genetic (upper matrix) and environmental correlations (lower matrix) of whole CC DTI measures and total WMLs for scan 2.
(DOC)

## References

[1] Van der Knaap LJ, van der Ham IJ (2011) How does the corpus callosum mediate interhemispheric transfer? A review. Behavioural Brain Research 223:211–221. 21530590 10.1016/j.bbr.2011.04.018

[2] Aboitiz F, Scheibel AB, Fisher RS, Zaidel E (1992) Fiber composition of the human corpus callosum. Brain Research 598:143–153. 1486477 10.1016/0006-8993(92)90178-c

[3] Bloom JS, Hynd GW (2005) The role of the corpus callosum in interhemispheric transfer of information: excitation or inhibition? Neuropsychology Review 15:59–71. 16211466 10.1007/s11065-005-6252-y

[4] Basser PJ, Mattiello J, LeBihan D (1994) MR diffusion tensor spectroscopy and imaging. Biophysical Journal 66:259–267. 8130344 10.1016/S0006-3495(94)80775-1 PMC1275686

[5] Persson J, Nyberg L, Lind J, Larsson A, Nilsson LG, et al (2006) Structure-function correlates of cognitive decline in aging. Cerebral Cortex 16:907–915. 16162855 10.1093/cercor/bhj036

[6] Bastin ME, Clayden JD, Pattie A, Gerrish IF, Wardlaw JM, et al (2009) Diffusion tensor and magnetization transfer MRI measurements of periventricular white matter hyperintensities in old age. Neurobiology of Aging 30:125–136. 17624630 10.1016/j.neurobiolaging.2007.05.013

[7] Beaulieu C (2002) The basis of anisotropic water diffusion in the nervous system - a technical review. NMR in Biomedicine 15:435–455. 12489094 10.1002/nbm.782

[8] Budde MD, Kim JH, Liang HF, Schmidt RE, Russell JH, et al (2007) Toward accurate diagnosis of white matter pathology using diffusion tensor imaging. Magnetic resonance in medicine: Official Journal of the Society of Magnetic Resonance in Medicine/Society of Magnetic Resonance in Medicine 57:688–695. 10.1002/mrm.21200 17390365

[9] Song SK, Sun SW, Ju WK, Lin SJ, Cross AH, et al (2003) Diffusion tensor imaging detects and differentiates axon and myelin degeneration in mouse optic nerve after retinal ischemia. NeuroImage 20:1714–1722. 14642481 10.1016/j.neuroimage.2003.07.005

[10] Song SK, Yoshino J, Le TQ, Lin SJ, Sun SW, et al (2005) Demyelination increases radial diffusivity in corpus callosum of mouse brain. NeuroImage 26:132–140. 15862213 10.1016/j.neuroimage.2005.01.028

[11] Schulte T, Sullivan EV, Muller-Oehring EM, Adalsteinsson E, Pfefferbaum A (2005) Corpus callosal microstructural integrity influences interhemispheric processing: a diffusion tensor imaging study. Cerebral Cortex 15:1384–1392. 15635059 10.1093/cercor/bhi020

[12] Luders E, Thompson PM, Toga AW (2010) The development of the corpus callosum in the healthy human brain. The Journal of Neuroscience 30:10985–10990. 20720105 10.1523/JNEUROSCI.5122-09.2010 PMC3197828

[13] Salat D, Ward A, Kaye JA, Janowsky JS (1997) Sex differences in the corpus callosum with aging. Neurobiology of Aging 18:191–197. 9258896 10.1016/s0197-4580(97)00014-6

[14] Takeda S, Hirashima Y, Ikeda H, Yamamoto H, Sugino M, et al (2003) Determination of indices of the corpus callosum associated with normal aging in Japanese individuals. Neuroradiology 45:513–518. 12879325 10.1007/s00234-003-1019-8

[15] Ota M, Obata T, Akine Y, Ito H, Ikehira H, et al (2006) Age-related degeneration of corpus callosum measured with diffusion tensor imaging. NeuroImage 31:1445–1452. 16563802 10.1016/j.neuroimage.2006.02.008

[16] Sullivan EV, Rohlfing T, Pfefferbaum A (2010) Longitudinal study of callosal microstructure in the normal adult aging brain using quantitative DTI fiber tracking. Developmental Neuropsychology 35:233–256. 20446131 10.1080/87565641003689556 PMC2867078

[17] Fling BW, Chapekis M, Reuter-Lorenz PA, Anguera J, Bo J, et al (2011) Age differences in callosal contributions to cognitive processes. Neuropsychologia 49:2564–2569. 21601582 10.1016/j.neuropsychologia.2011.05.004 PMC3137668

[18] Ryberg C, Rostrup E, Paulson OB, Barkhof F, Scheltens P, et al (2011) Corpus callosum atrophy as a predictor of age-related cognitive and motor impairment: a 3-year follow-up of the LADIS study cohort. Journal of the Neurological Sciences 307:100–105. 21621224 10.1016/j.jns.2011.05.002

[19] Zahr NM, Rohlfing T, Pfefferbaum A, Sullivan EV (2009) Problem solving, working memory, and motor correlates of association and commissural fiber bundles in normal aging: a quantitative fiber tracking study. NeuroImage 44:1050–1062. 18977450 10.1016/j.neuroimage.2008.09.046 PMC2632960

[20] Salami A, Eriksson J, Nilsson LG, Nyberg L (2012) Age-related white matter microstructural differences partly mediate age-related decline in processing speed but not cognition. Biochimica et Biophysica Acta 1822:408–415. 21930202 10.1016/j.bbadis.2011.09.001

[21] Serbruyns L, Gooijers J, Caeyenberghs K, Meesen RL, Cuypers K, et al. (2013) Bimanual motor deficits in older adults predicted by diffusion tensor imaging metrics of corpus callosum subregions. Brain Structure & Function. 10.1007/s00429-013-0654-z 24158531

[22] Di Paola M, Luders E, Di Iulio F, Cherubini A, Passafiume D, et al (2010) Callosal atrophy in mild cognitive impairment and Alzheimer's disease: different effects in different stages. NeuroImage 49:141–149. 19643188 10.1016/j.neuroimage.2009.07.050 PMC2764791

[23] Filippini N, Douaud G, Mackay CE, Knight S, Talbot K, et al (2010) Corpus callosum involvement is a consistent feature of amyotrophic lateral sclerosis. Neurology 75:1645–1652. 21041787 10.1212/WNL.0b013e3181fb84d1 PMC2974368

[24] Wang PJ, Saykin AJ, Flashman LA, Wishart HA, Rabin LA, et al (2006) Regionally specific atrophy of the corpus callosum in AD, MCI and cognitive complaints. Neurobiology of aging 27:1613–1617. 16271806 10.1016/j.neurobiolaging.2005.09.035 PMC3482483

[25] Wiltshire K, Concha L, Gee M, Bouchard T, Beaulieu C, et al (2010) Corpus callosum and cingulum tractography in Parkinson's disease. The Canadian Journal of Neurological Sciences 37:595–600. 21059504 10.1017/s0317167100010751

[26] Alves GS, O'Dwyer L, Jurcoane A, Oertel-Knochel V, Knochel C, et al (2012) Different patterns of white matter degeneration using multiple diffusion indices and volumetric data in mild cognitive impairment and Alzheimer patients. PloS one 7:e52859. 23300797 10.1371/journal.pone.0052859 PMC3534120

[27] Duffy SL, Paradise M, Hickie IB, Lewis SJ, Naismith SL, et al (2013) Cognitive impairment with and without depression history: an analysis of white matter microstructure. Journal of Psychiatry & Neuroscience 38:130079. 10.1503/jpn.130079 PMC3937282 24359878

[28] Liu J, Yin C, Xia S, Jia L, Guo Y, et al (2013) White matter changes in patients with amnestic mild cognitive impairment detected by diffusion tensor imaging. PloS one 8:e59440. 23555673 10.1371/journal.pone.0059440 PMC3605411

[29] Wang JH, Lv PY, Wang HB, Li ZL, Li N, et al (2013) Diffusion tensor imaging measures of normal appearing white matter in patients who are aging, or have amnestic mild cognitive impairment, or Alzheimer's disease. Journal of Clinical Neuroscience 20:1089–1094. 23787190 10.1016/j.jocn.2012.09.025

[30] Kanchibhotla SC, Mather KA, Wen W, Schofield PR, Kwok JB, et al (2013) Genetics of ageing-related changes in brain white matter integrity - a review. Ageing Research Reviews 12:391–401. 23128052 10.1016/j.arr.2012.10.003

[31] Pfefferbaum A, Sullivan EV, Carmelli D (2001) Genetic regulation of regional microstructure of the corpus callosum in late life. Neuroreport 12:1677–1681. 11409738 10.1097/00001756-200106130-00032

[32] Kochunov P, Glahn DC, Lancaster JL, Winkler AM, Smith S, et al (2010) Genetics of microstructure of cerebral white matter using diffusion tensor imaging. NeuroImage 53:1109–1116. 20117221 10.1016/j.neuroimage.2010.01.078 PMC2888778

[33] Aboitiz F, Scheibel AB, Fisher RS, Zaidel E (1992) Individual differences in brain asymmetries and fiber composition in the human corpus callosum. Brain Research 598:154–161. 1486478 10.1016/0006-8993(92)90179-d

[34] Clarke JM, Zaidel E (1994) Anatomical-behavioral relationships: corpus callosum morphometry and hemispheric specialization. Behavioural Brain Research 64:185–202. 7840886 10.1016/0166-4328(94)90131-7

[35] Hasan KM, Kamali A, Kramer LA, Papnicolaou AC, Fletcher JM, et al (2008) Diffusion tensor quantification of the human midsagittal corpus callosum subdivisions across the lifespan. Brain Research 1227:52–67. 18598682 10.1016/j.brainres.2008.06.030 PMC2602603

[36] Witelson SF (1989) Hand and sex differences in the isthmus and genu of the human corpus callosum. A postmortem morphological study. Brain 112 (Pt 3):799–835. 2731030 10.1093/brain/112.3.799

[37] Stievenart JL, Iba-Zizen MT, Tourbah A, Lopez A, Thibierge M, et al (1997) Minimal surface: a useful paradigm to describe the deeper part of the corpus callosum? Brain Research Bulletin 44:117–124. 9292200 10.1016/s0361-9230(97)00113-5

[38] Weis S, Kimbacher M, Wenger E, Neuhold A (1993) Morphometric analysis of the corpus callosum using MR: correlation of measurements with aging in healthy individuals. AJNR American Journal of Neuroradiology 14:637–645. 8517352 PMC8333387

[39] Chao YP, Cho KH, Yeh CH, Chou KH, Chen JH, et al (2009) Probabilistic topography of human corpus callosum using cytoarchitectural parcellation and high angular resolution diffusion imaging tractography. Human Brain Mapping 30:3172–3187. 19241418 10.1002/hbm.20739 PMC6871153

[40] Hofer S, Frahm J (2006) Topography of the human corpus callosum revisited—comprehensive fiber tractography using diffusion tensor magnetic resonance imaging. NeuroImage 32:989–994. 16854598 10.1016/j.neuroimage.2006.05.044

[41] Huang H, Zhang J, Jiang H, Wakana S, Poetscher L, et al (2005) DTI tractography based parcellation of white matter: application to the mid-sagittal morphology of corpus callosum. NeuroImage 26:195–205. 15862219 10.1016/j.neuroimage.2005.01.019

[42] Park HJ, Kim JJ, Lee SK, Seok JH, Chun J, et al (2008) Corpus callosal connection mapping using cortical gray matter parcellation and DT-MRI. Human Brain Mapping 29:503–516. 17133394 10.1002/hbm.20314 PMC6870924

[43] Fazekas F (1989) Magnetic resonance signal abnormalities in asymptomatic individuals: their incidence and functional correlates. European Neurology 29:164–168. 2731564 10.1159/000116401

[44] Wen W, Sachdev P (2004) The topography of white matter hyperintensities on brain MRI in healthy 60- to 64-year-old individuals. NeuroImage 22:144–154. 15110004 10.1016/j.neuroimage.2003.12.027

[45] Assareh A, Mather KA, Schofield PR, Kwok JB, Sachdev PS (2011) The genetics of white matter lesions. CNS Neuroscience & Therapeutics 17:525–540. 21951372 10.1111/j.1755-5949.2010.00181.x PMC6493881

[46] de Groot M, Verhaaren BF, de Boer R, Klein S, Hofman A, et al (2013) Changes in normal-appearing white matter precede development of white matter lesions. Stroke 44:1037–1042. 23429507 10.1161/STROKEAHA.112.680223

[47] Iverson GL, Hakulinen U, Waljas M, Dastidar P, Lange RT, et al (2011) To exclude or not to exclude: white matter hyperintensities in diffusion tensor imaging research. Brain Injury 25:1325–1332. 22077537 10.3109/02699052.2011.608409

[48] Lange RT, Shewchuk JR, Heran MK, Rauscher A, Jarrett M, et al (2013) To Exclude or Not To Exclude: Further Examination of the Influence of White Matter Hyperintensities in Diffusion Tensor Imaging Research. Journal of Neurotrauma 31(2):198–205. 23952763 10.1089/neu.2013.2866

[49] Sachdev PS, Lammel A, Trollor JN, Lee T, Wright MJ, et al (2009) A comprehensive neuropsychiatric study of elderly twins: the Older Australian Twins Study. Twin Research and Human Genetics 12:573–582. 19943720 10.1375/twin.12.6.573

[50] Batouli SA, Sachdev PS, Wen W, Wright MJ, Suo C, et al (2012) The heritability of brain metabolites on proton magnetic resonance spectroscopy in older individuals. NeuroImage 62:281–289. 22561359 10.1016/j.neuroimage.2012.04.043

[51] Behrens TE, Woolrich MW, Jenkinson M, Johansen-Berg H, Nunes RG, et al (2003) Characterization and propagation of uncertainty in diffusion-weighted MR imaging. Magnetic Resonance in Medicine 50:1077–1088. 14587019 10.1002/mrm.10609

[52] Jenkinson M, Beckmann CF, Behrens TE, Woolrich MW, Smith SM (2012) FSL. NeuroImage 62:782–790. 21979382 10.1016/j.neuroimage.2011.09.015

[53] Wen W, Sachdev PS, Li JJ, Chen X, Anstey KJ (2009) White matter hyperintensities in the forties: their prevalence and topography in an epidemiological sample aged 44–48. Human Brain Mapping 30:1155–1167. 18465744 10.1002/hbm.20586 PMC6870596

[54] Williams VJ, Leritz EC, Shepel J, McGlinchey RE, Milberg WP, et al (2013) Interindividual variation in serum cholesterol is associated with regional white matter tissue integrity in older adults. Human Brain Mapping 34:1826–1841. 22438182 10.1002/hbm.22030 PMC3707964

[55] Burgmans S, van Boxtel M, Gronenschild E, Vuurman E, Hofman P, et al (2010) Multiple Indicators of Age-related Differences in Cerebral White Matter and the Modifying Effects of Hypertension. NeuroImage 49:2083. 19850136 10.1016/j.neuroimage.2009.10.035 PMC2818314

[56] Maillard P, Seshadri S, Beiser A, Himali JJ, Au R, et al (2012) Effects of systolic blood pressure on white-matter integrity in young adults in the Framingham Heart Study: a cross-sectional study. Lancet Neurology 11:1039–1047. 23122892 10.1016/S1474-4422(12)70241-7 PMC3510663

[57] McQueeny T, Schweinsburg BC, Schweinsburg AD, Jacobus J, Bava S, et al (2009) Altered white matter integrity in adolescent binge drinkers. Alcoholism, Clinical and Experimental research 33:1278–1285. 10.1111/j.1530-0277.2009.00953.x PMC2825379 19389185

[58] Hudkins M, O'Neill J, Tobias MC, Bartzokis G, London ED (2012) Cigarette smoking and white matter microstructure. Psychopharmacology 221:285–295. 22215225 10.1007/s00213-011-2621-9 PMC4111107

[59] Antenor-Dorsey JA, Meyer E, Rutlin J, Perantie DC, White NH, et al (2013) White matter microstructural integrity in youth with type 1 diabetes. Diabetes 62:581–589. 23139349 10.2337/db12-0696 PMC3554385

[60] Reijmer YD, Brundel M, de Bresser J, Kappelle LJ, Leemans A, et al (2013) Microstructural white matter abnormalities and cognitive functioning in type 2 diabetes: a diffusion tensor imaging study. Diabetes Care 36:137–144. 22961577 10.2337/dc12-0493 PMC3526236

[61] Almasy L, Blangero J (1998) Multipoint quantitative-trait linkage analysis in general pedigrees. American Journal of Human Genetics 62:1198–1211. 9545414 10.1086/301844 PMC1377101

[62] Visscher PM, Gordon S, Neale MC (2008) Power of the classical twin design revisited: II detection of common environmental variance. Twin Research and Human Genetics 11:48–54. 18251675 10.1375/twin.11.1.48 PMC3996914

[63] Jahanshad N, Kochunov PV, Sprooten E, Mandl RC, Nichols TE, et al (2013) Multi-site genetic analysis of diffusion images and voxelwise heritability analysis: a pilot project of the ENIGMA-DTI working group. NeuroImage 81:455–469. 23629049 10.1016/j.neuroimage.2013.04.061 PMC3729717

[64] Almasy L, Dyer TD, Blangero J (1997) Bivariate quantitative trait linkage analysis: pleiotropy versus co-incident linkages. Genetic Epidemiology 14:953–958. 9433606 10.1002/(SICI)1098-2272(1997)14:6<953::AID-GEPI65>3.0.CO;2-K

[65] Hopper JL, Mathews JD (1982) Extensions to multivariate normal models for pedigree analysis. Annals of Human Genetics 46:373–383. 6961886 10.1111/j.1469-1809.1982.tb01588.x

[66] Williams JT, Van Eerdewegh P, Almasy L, Blangero J (1999) Joint multipoint linkage analysis of multivariate qualitative and quantitative traits. I. Likelihood formulation and simulation results. American Journal of Human Genetics 65:1134–1147. 10486333 10.1086/302570 PMC1288247

[67] Alger JR (2012) The diffusion tensor imaging toolbox. J Neurosci 32:7418–7428. 22649222 10.1523/JNEUROSCI.4687-11.2012 PMC3444512

[68] Seiler S, Cavalieri M, Schmidt R (2012) Vascular cognitive impairment - An ill-defined concept with the need to define its vascular component. J Neurol Sci 22:22. 10.1016/j.jns.2012.06.001 22727978

[69] Fazekas F, Wardlaw JM (2013) The origin of white matter lesions: a further piece to the puzzle. Stroke 44:951–952. 23429508 10.1161/STROKEAHA.111.000849

[70] Srikanth V, Phan TG, Chen J, Beare R, Stapleton JM, et al (2010) The location of white matter lesions and gait–a voxel-based study. Annals of Neurology 67:265–269. 20225293 10.1002/ana.21826

