# Supplementary material for: Genetics of Microstructure of the Corpus Callosum in Older Adults
Source: PLoS One. 2014 Dec 16;9(12):e113181. doi: 10.1371/journal.pone.0113181 (PMC4267776; doi:10.1371/journal.pone.0113181)
Supplement: S1 File — This file contains S1 Table–S5 Table. S1a Table, Summary of covariate results used in the variance components model for whole CC and its sub regions (FA & MD). S1b Table, Summary of covariate results used in the variance components model for whole CC and its sub regions (RD & AD). S2a Table, Intra-class correlations (ICC) estimates for MZ and DZ twin groups for whole CC DTI measures for Scan 1. S2b Table, Intra-class correlations (ICC) estimates for MZ and DZ twin groups for whole CC DTI measures for Scan 2. S3a Table, Heritability estimates for Whole CC and its five sub-regions [A–E] for the four DTI measures for scan 1. S3b Table, Heritability estimates for Whole CC and its five sub-regions [A–E] for the four DTI measures for scan 2. S4 Table, Heritability estimates for the four DTI measures of whole CC in females. S5a Table, Genetic (upper matrix) and environmental correlations (lower matrix) of whole CC DTI measures and total WMLs for scan 1. S5b Table, Genetic (upper matrix) and environmental correlations (lower matrix) of whole CC DTI measures and total WMLs for scan 2. (DOC) [file pone.0113181.s001.doc]

**Table S1a: Summary of covariate results used in the variance components model for whole CC and its sub regions** (FA & MD)

|  | **FA** | | | | | | **MD** | | | | | |
| --- | --- | --- | --- | --- | --- | --- | --- | --- | --- | --- | --- | --- |
|  | W_CC | A | B | C | D | E | W_CC | A | B | C | D | E |
| Age_β value (SE) | -0.03 (0.01) | -0.04 (0.01) | -0.02 (0.01) | -0.02 (0.01) | -0.04 (0.01) | -0.03 0.01) | 0.05 (0.01) | 0.05 (0.01) | 0.04 (0.01) | 0.02 (0.01) | 0.03 (0.01) | 0.03 (0.01) |
| Age_p-value | 0.01 | 0.01 | 0.15 | 0.25 | 0.002 | 0.04 | 1.46 x 10^-6^ | 1.06 x 10^-6^ | 9.64 x 10^-5^ | 0.04 | 0.003 | 0.3 x 10^-3^ |
| Sex_ β value (SE) | 0.06 (0.12) | 0.06 (0.12) | 0.03 (0.13) | 0.18 (0.13) | 0.27 (0.12) | 0.02 (0.12) | -0.24 (0.09) | -0.27 (0.10) | -0.34 (0.10) | -0.11 (0.12) | -0.16 (0.10) | -0.10 (0.08) |
| Sex_p-value | 0.65 | 0.61 | 0.84 | 0.17 | 0.03 | 0.85 | 0.009 | 0.008 | 0.001 | 0.37 | 0.13 | 0.25 |
| Scanner_1_ β value (SE) | 0.94 (0.15) | 0.88 (0.15) | 0.85 (0.16) | 0.70 (0.16) | 0.94 (0.15) | 0.57 (0.15) | -1.49 (0.11) | -1.20 (0.12) | -1.26 (0.13) | -0.96 (0.14) | -1.56 (0.12) | -1.43 (0.10) |
| Scanner_1_p-value | 2.47 x 10^-9^ | 2.68 x 10^-8^ | 2.23 x 10^-7^ | 0.14 x 10^-4^ | 1.05 x 10^-9^ | 0.27 x 10^-3^ | 7.86 x 10^--31^ | 1.39 x 10^-18^ | 1.56 x 10^-18^ | 3.04 x 10^-10^ | 9.21 x 10^-27^ | 8.02 x 10^-31^ |
| Scanner_2_ β value (SE) | 0.70 (0.16) | 0.57 (0.16) | 0.35 (0.16) | 0.37 (0.16) | 0.77 (0.15) | 0.65 (0.16) | 0.10 (0.11) | 0.26 (0.13) | 0.02 (0.13) | 0.05 (0.15) | -0.71 (0.13) | 0.27 (0.11) |
| Scanner_2_p-value | 2.15 x 10^-5^ | 0.53 x 10^-3^ | 0.03 | 0.02 | 1.34 x 10^-6^ | 7.56 x 10^-5^ | 0.37 | 0.04 | 0.87 | 0.73 | 3.28 x 10^-7^ | 0.01 |
| Scanner_3_ β value (SE) | 1.17 (0.22) | 1.22 (0.22) | 0.35 (0.23) | 0.33 (0.23) | 0.90 (0.22) | 1.19 (0.22) | -0.95 (0.16) | -0.74 (0.18) | -0.62 (0.19) | -0.39 (0.21) | -1.37 (0.18) | -0.95 (0.15) |
| Scanner_3_p-value | 2.42 x 10^-7^ | 1.24 x 10^-7^ | 0.13 | 0.15 | 5.59 x 10^-5^ | 3.06 x 10^-7^ | 1.23 x 10^-8^ | 0.66 x 10^-4^ | 0.001 | 0.07 | 4.66 x 10^-12^ | 5.64 x 10^-9^ |
| Handedness_β value (SE) | -0.1 x 10^-2^ (0.9 x 10^-3^) | -0.1 x 10^-2^ (0.9 x 10^-3^) | -0.1 x 10^-2^ (0.9 x 10^-3^) | -0.1 x 10^-2^ (0.9 x 10^-3^) | -0.9 x 10^-3^ (0.9 x 10^-3^) | -0.1 x 10^-2^ (0.9 x 10^-3^) | 0.001 (0.6 x 10^-3^) | 0.13 x 10^-2^ (0.7 x 10^-3^) | 0.18 x 10^-2^ (0.7 x 10^-3^) | 0.17 x 10^-2^ (0.9 x 10^-3^) | 0.12 x 10^-2^ (0.7 x 10^-3^) | 0.15 x 10^-2^ (0.6 x 10^-3^) |
| Handedness_p-value | 0.10 | 0.17 | 0.26 | 0.27 | 0.35 | 0.15 | 0.006 | 0.07 | 0.01 | 0.04 | 0.11 | 0.01 |
| Diastolic mean_ β value (SE) | 0.01 (0.7 x 10^-2^) | 0.77 x 10^-2^ (0.7 x 10^-2^) | 0.61 x 10^-2^ (0.8 x 10^-2^ ) | 0.76 x 10^-2^ (0.8 x 10^-2^) | 0.45 x 10^-2^ (0.8 x 10^-2^) | 0.01 (0.7 x 10^-2^) | -0.30 x 10^-3^ (0.5 x 10^-2^) | 0.53 x 10^-2^ (0.6 x 10^-2^) | -0.45 x 10^-2^ (0.6 x 10^-2^) | -0.01 (0.7 x 10^-2^) | -0.50 x 10^-2^ (0.60 x 10^-2^) | 0.6 0x 10^-3^ (0.5 x 10^-2^) |
| Diastolic mean_p-value | 0.08 | 0.29 | 0.43 | 0.34 | 0.54 | 0.009 | 0.94 | 0.37 | 0.47 | 0.06 | 0.43 | 0.90 |
| Systolic mean_β value (SE) | -0.7 x 10^-2^ (0.42 x 10^-2^) | -0.01 (0.4 x 10^-2^) | -0.5 x 10^-2^ (0.46 x 10^-2^ ) | -0.5 x 10^-2^ (0.47 x 10^-2^) | -0.2 x 10^-2^ (0.44 x 10^-2^) | -0.11 x 10^-1^ (0.43 x 10^-2^) | 0.19 x 10^-2^ (0.31 x 10^-2^) | -0.17 x 10^-2^ (0.35 x 10^-2^) | 0.27 x 10^-2^ (0.36 x 10^-2^ ) | 0.54 x 10^-2^ (0.42 x 10^-2^ ) | 0.31 x 10^-2^ (0.37 x 10^-2^) | 0.33 x 10^-2^ (0.30 x 10^-2^) |
| Systolic mean_p-value | 0.06 | 0.18 | 0.27 | 0.32 | 0.61 | 0.01 | 0.54 | 0.62 | 0.45 | 0.19 | 0.39 | 0.26 |

Notes: FA – Fractional anisotropy; MD- Mean diffusivity; SE – standard error.

**Table S1b: Summary of covariate results used in the variance components model for whole CC and its sub regions** (RD & AD)

|  | **RD** | | | | | | **AD** | | | | | |
| --- | --- | --- | --- | --- | --- | --- | --- | --- | --- | --- | --- | --- |
|  | W_CC | A | B | C | D | E | W_CC | A | B | C | D | E |
| Age_β value (SE) | 0.05 (0.01) | 0.06 (0.01) | 0.04 (0.01) | 0.02 (0.01) | 0.04 (0.01) | 0.04 (0.01) | 0.04 (0.09 x 10^-1^ ) | 0.04 (0.01) | 0.03 (0.01) | 0.02 (0.01) | 0.02 (0.01) | 0.02 (0.09 x 10^-1^ ) |
| Age_p-value | 5.00 x 10^-6^ | 2.23 x 10^-6^ | 0.03 x 10^-2^ | 0.06 | 0.001 | 0.2 x 10^-3^ | 0.01 x 10^-2^ | 0.01 x 10^-2^ | 0.02 x 10^-1^ | 0.02 | 0.04 | 0.01 |
| Sex_ β value (SE) | -0.2 (0.10) | -0.22 (0.11) | -0.27 (0.12) | -0.12 (0.13) | -0.2 (0.11) | -0.1 (0.10) | 0.19 (0.09) | 0.18 (0.10) | 0.25 (0.09) | 0.14 (0.10) | 0.19 (0.10) | 0.04 (0.09) |
| Sex_p-value | 0.06 | 0.05 | 0.02 | 0.35 | 0.08 | 0.36 | 0.03 | 0.07 | 0.08x10^-1^ | 0.18 | 0.06 | 0.66 |
| Scanner_1_ β value (SE) | -1.19 (0.13) | -0.93 (0.13) | -1.02 (0.14) | -0.75 (0.15) | -1.3 (0.13) | -1.11 (0.12) | -1.57 (0.10) | -1.27 (0.12) | -0.99 (0.11) | -0.92 (0.12) | -1.38 (0.12) | -1.43 (0.11) |
| Scanner_1_p-value | 2.89 x 10^-17^ | 1.29 x 10^-10^ | 2.20 x 10^-11^ | 2.68 x 10^-6^ | 4.12 x 10^-18^ | 6.00 x 10^-16^ | 9.07 x 10^-33^ | 3.08 x 10^-20^ | 1.52 x 10^-15^ | 2.17 x 10^-11^ | 4.62 x 10^-22^ | 1.35 x 10^-27^ |
| Scanner_2_ β value (SE) | -0.08 (0.13) | 0.11 (0.14) | -0.04 (0.15) | -0.01 (0.16) | -0.76 (0.14) | 0.03 (0.13) | -0.01 (0.11) | -0.04 (0.12) | 0.17 (0.11) | -0.07 (0.13) | -0.49 (0.12) | 0.11 (0.11) |
| Scanner_2_p-value | 0.56 | 0.43 | 0.78 | 0.95 | 3.60 x 10^-7^ | 0.81 | 0.91 | 0.75 | 0.13 | 0.59 | 0.01 x 10^-2^ | 0.32 |
| Scanner_3_ β value (SE) | -1.2 (0.19) | -1.01 (0.20) | -0.72 (0.21) | -0.46 (0.23) | -1.4 (0.20) | -1.3 (0.19) | -0.71 (0.16) | -0.47 (0.18) | -0.49 (0.17) | -0.29 (0.19) | -1.08 (0.18) | -0.60 (0.16) |
| Scanner_3_p-value | 6.07 x 10^-10^ | 1.29 x 10^-6^ | 0.83 x 10^-3^ | 0.04 | 5.30 x 10^-11^ | 6.19 x 10^-11^ | 0.01 x 10^-3^ | 0.01 | 0.04 x 10^-1^ | 0.12 | 1.88 x 10^-8^ | 0.03 x 10^-2^ |
| Handedness_β value (SE) | 0.16 x 10^-2^ (0.7 x 10^-3^) | 0.12 x 10^-2^ (0.8 x 10^-3^) | 0.16 x 10^-2^ (0.08 x 10^-2^) | 0.15 x 10^-2^ (0.09 x 10^-2^) | 0.1 x 10^-2^ (0.08 x 10^-2^) | 0.15 x 10^-2^ (0.7 x 10^-3^) | 0.01 x 10^-1^ (0.06 x 10^-2^ ) | 0.99 x 10^-3^ (0.07 x 10^-2^ ) | 0.01 x 10^-1^ (0.07 x 10^-2^ ) | 0.15 x 10^-2^ (0.08 x 10^-2^ ) | 0.09 x 10^-2^ (0.07 x 10^-2^ ) | 0.13 x 10^-2^ (0.06 x 10^-2^ ) |
| Handedness_p-value | 0.02 | 0.12 | 0.04 | 0.1 | 0.2 | 0.05 | 0.01 | 0.17 | 0.07 | 0.06 | 0.24 | 0.06 |
| Diastolic mean_ β value (SE) | -0.35 x 10^-2^ (0.6 x 10^-2^) | 0.36 x 10^-2^ (0.7 x 10^-2^ ) | -0.49 x 10^-2^ (0.7 x 10^-2^ ) | -0.01 (0.8 x 10^-2^ ) | -0.56 x 10^-2^ (0.7 x 10^-2^ ) | -0.66 x 10^-2^ (0.6 x 10^-2^ ) | 0.07 x 10^-1^ (0.54 x 10^-2^ ) | 0.06 x 10^-1^ (0.62 x 10^-2^ ) | 0.04 x 10^-1^ (0.05 x 10^-1^ ) | -0.49 x 10^-2^ (0.06 x 10^-1^ ) | 0.02 x 10^-1^ (0.06 x 10^-1^ ) | 0.09 x 10^-1^ (0.05 x 10^-1^ ) |
| Diastolic mean_p-value | 0.57 | 0.58 | 0.48 | 0.1 | 0.41 | 0.3 | 0.19 | 0.27 | 0.46 | 0.47 | 0.68 | 0.09 |
| Systolic mean_β value (SE) | 0.38 x 10^-2^ (0.36 x 10^-2^) | -0.35 x 10^-3^ (0.4 x 10^-2^) | 0.39 x 10^-2^ (0.41 x 10^-2^) | 0.62 x 10^-2^ (0.45 x 10^-2^) | 0.35 x 10^-2^ (0.4 x 10^-2^) | 0.7 x 10^-2^ (0.37 x 10^-2^) | -0.02 x 10^-1^ (0.31 x 10^-2^) | -0.22 x 10^-2^ (0.36 x 10^-2^) | -0.03 x 10^-1^ (0.34 x 10^-2^) | -0.04 x 10^-2^ (0.39 x 10^-2^) | 0.16 x 10^-2^ (0.03 x 10^-1^) | -0.01 x 10^-1^ (0.03 x 10^-1^) |
| Systolic mean_p-value | 0.29 | 0.92 | 0.34 | 0.16 | 0.37 | 0.05 | 0.49 | 0.53 | 0.32 | 0.9 | 0.66 | 0.65 |

Notes: RD- radial diffusivity; AD – Axial diffusivity; SE – standard error

**Table S2a: Intra-class correlations (ICC) estimates for MZ and DZ twin groups for whole CC DTI measures for Scan 1**

| **Whole CC** | **MZ ICC (95% C.I.)** | **DZ ICC (95% C.I.)** |
| --- | --- | --- |
| FA | 0.63 (0.48-0.75) | 0.33 (0.09-0.53) |
| MD | 0.72 (0.60-0.81) | 0.60 (0.42-0.74) |
| RD | 0.58 (0.41-0.71) | 0.33 (0.09-0.54) |
| AD | 0.64 (0.49-0.75) | 0.70 (0.54-0.80) |

Notes: MZ – monozygotic twins; DZ- Dizygotic twins; FA – Fractional anisotropy; MD- Mean diffusivity; RD- radial diffusivity; AD – Axial diffusivity; ICC- Intra-class correlations

**Table S2b: Intra-class correlations (ICC) estimates for MZ and DZ twin groups for whole CC DTI measures for Scan 2**

| **Whole CC** | **MZ ICC (95% C.I.)** | **DZ ICC (95% C.I.)** |
| --- | --- | --- |
| FA | 0.51 (0.32-0.65) | 0.40 (0.17-0.59) |
| MD | 0.73 (0.60-0.82) | 0.64 (0.46-0.76) |
| RD | 0.64 (0.49-0.76) | 0.54 (0.34-0.70) |
| AD | 0.58 (0.41-0.71) | 0.73 (0.59-0.83) |

Notes: MZ – monozygotic twins; DZ- Dizygotic twins; FA – Fractional anisotropy; MD- Mean diffusivity; RD- radial diffusivity; AD – Axial diffusivity; ICC- Intra-class correlations

**Table S3**a: Heritability estimates for Whole CC and its five sub-regions [A-E] for the four DTI measures for scan 1

| **CC Region** | **FA** | | **MD** | | **RD** | | **AD** | |
| --- | --- | --- | --- | --- | --- | --- | --- | --- |
|  | **h^2^ ± SE** | **p-value** | **h^2^ ± SE** | **p-value** | **h^2^ ± SE** | **p-value** | **h^2^ ± SE** | **p-value** |
| Whole CC | 0.55 ± 0.07 | 6.80 x 10^-10*^ | 0.45 ± 0.09 | 0.10 x 10^-4*^ | 0.41 ± 0.09 | 0.16 x 10^-4*^ | 0.30± 0.09 | 1.03 x 10^-3*^ |
| A | 0.53 ± 0.07 | 1.07 x 10^-8*^ | 0.37 ± 0.09 | 0.26 x 10^-3*^ | 0.33 ± 0.09 | 0.62 x 10^-3*^ | 0.23 ± 0.11 | 0.02 |
| B | 0.43 ± 0.08 | 1.50 x 10^-6*^ | 0.43 ± 0.10 | 0.53 x 10^-4*^ | 0.47 ± 0.09 | 0.02 x 10^-4*^ | 0.28 ± 0.09 | 0.28 x10^-2*^ |
| C | 0.24 ± 0.10 | 0.78 x 10^-2*^ | 0.23 ± 0.10 | 0.11 x 10^-1*^ | 0.27 ± 0.09 | 2.60 x 10^-3*^ | 0.009 ± 0.10 | 0.46 |
| D | 0.30 ± 0.09 | 0.51 x 10^-3*^ | 0.38 ± 0.09 | 0.38 x 10^-4*^ | 0.40 ± 0.08 | 0.11 x 10^-4*^ | 0.08 ± 0.10 | 0.22 |
| E | 0.49 ± 0.07 | 1.00 x 10^-7*^ | 0.35 ± 0.09 | 0.25 x 10^-3*^ | 0.32 ± 0.09 | 0.46x 10^-3*^ | 0.20 ±0.10 | 0.02 |

^*^Values are significant at p<.0001

Notes: FA – Fractional anisotropy; MD- Mean diffusivity; RD- radial diffusivity; AD – Axial diffusivity; h^2^- heritability estimate; SE – standard error.

**Table S3**b: Heritability estimates for Whole CC and its five sub-regions [A-E] for the four DTI measures for scan 2

| **CC Region** | **FA** | | **MD** | | **RD** | | **AD** | |
| --- | --- | --- | --- | --- | --- | --- | --- | --- |
|  | **h^2^ ± SE** | **p-value** | **h^2^ ± SE** | **p-value** | **h^2^ ± SE** | **p-value** | **h^2^ ± SE** | **p-value** |
| Whole CC | 0.44 ± 0.07 | 3.00 x 10^-7*^ | 0.52 ± 0.08 | 0.30 x 10^-6*^ | 0.48 ± 0.08 | 0.20 x 10^-6*^ | 0.39 ± 0.08 | 2.22 x 10^-5*^ |
| A | 0.44 ± 0.08 | 1.40 x 10^-6*^ | 0.46 ± 0.10 | 0.23 x 10^-4*^ | 0.43 ± 0.09 | 0.26 x 10^-4*^ | 0.17 ± 0.10 | 0.04 |
| B | 0.30 ± 0.08 | 0.50 x 10^-3*^ | 0.37 ± 0.09 | 0.17 x 10^-3*^ | 0.39 ± 0.08 | 0.20 x 10^-4*^ | 0.03 ± 0.08 | 0.33 |
| C | 0.34 ± 0.08 | 0.14 x 10^-3*^ | 0.36 ± 0.09 | 0.10 x 10^-3*^ | 0.39 ± 0.08 | 0.15 x 10^-4*^ | 0.05 ± 0.09 | 0.31 |
| D | 0.25 ± 0.09 | 0.28 x 10^-2*^ | 0.27 ± 0.09 | 0.20 x 10^-2*^ | 0.29 ± 0.09 | 0.72 x 10^-3*^ | 0.10 ± 0.10 | 0.15 |
| E | 0.40 ± 0.08 | 0.05 x 10^-4*^ | 0.37 ± 0.08 | 0.55 x 10^-4*^ | 0.41 ± 0.07 | 0.13 x 10^-5*^ | 0.09 ± 0.10 | 0.18 |

^*^Values are significant at p<.0001

Notes: FA – Fractional anisotropy; MD- Mean diffusivity; RD- radial diffusivity; AD – Axial diffusivity; h^2^- heritability estimate; SE – standard error

**Table S4 : Heritability estimates for the four DTI measures of whole CC in females.**

| **DTI measure** | **Whole_CC** | |
| --- | --- | --- |
|  | **h^2^ ± SE** | **p-value** |
| FA | 0.68 ± 0.07 | 1.83 x 10^-9*^ |
| MD | 0.58 ± 0.08 | 0.03 x 10^-5*^ |
| RD | 0.54 ± 0.09 | 0.07 x 10^-5*^ |
| AD | 0.39 ± 0.10 | 0.04 x 10^-2*^ |

^*^Values are significant at p<.0001

Notes: FA – Fractional anisotropy; MD- Mean diffusivity; RD- radial diffusivity; AD – Axial diffusivity; h^2^- heritability estimate; SE – standard error

**Table S5a: Genetic (upper matrix) and environmental correlations (lower matrix) of whole CC DTI measures and total WMLs for scan 1**

|  | **FA (ρ_g_ ± SE) [p]** | **MD (ρ_g_ ± SE) [p]** | **RD (ρ_g_ ± SE) [p]** | **AD (ρ_g_ ± SE) [p]** | **WMLs (ρ_g_ ± SE) [p]** |
| --- | --- | --- | --- | --- | --- |
| **FA (ρ_e_ ± SE) [p]** | 1 | -0.33 ± 0.12 [0.03] | -0.79 ± 0.07 [4.25 x 10^-6^]* | 0.22 ± 0.17 [0.19] | -0.28 ± 0.11 [0.02] |
| **MD (ρ_e_ ± SE) [p]** | -0.62 ± 0.07 [3.27 x 10^-10^]* | 1 | 0.93 ± 0.03 [ 0.31 x 10^-4^]* | 0.87 ± 0.09 [0.10 x 10^-3^] | 0.55 ± 0.15 [0.10 x 10^-3^]* |
| **RD (ρ_e_ ± SE) [p]** | -0.66 ± 0.06 [4.87 x 10^-15^]* | 0.85 ± 0.03 [3.32 x 10^-45^]* | 1 | 0.60 ± 0.15 [0.38 x10^-2^ ] | 0.53 ± 0.15 [0.19 x 10^-3^]* |
| **AD (ρ_e_ ± SE) [p]** | -0.10 ± 0.10 [ 0.34] | 0.53 ± 0.07 [3.07 x 10^-10^]* | 0.23 ± 0.09 [0.02] | 1 | 0.45 ± 0.14 [0.25 x 10^-2^] |
| **WMLs (ρ_e_ ± SE) [p]** | 0.10 ± 0.11 [0.34] | -0.17 ± 0.11 [0.14] | -0.14 ± 0.11 [0.18] | 0.03 ± 0.10 [0.74] | 1 |

^*^Values are significant at p<0.01.

Notes: FA – Fractional anisotropy; MD- Mean diffusivity; RD- radial diffusivity; AD – Axial diffusivity; WMLs – White matter lesions; ρ_g_ – genetic correlation coefficient; ρ_e_ - environmental correlations; SE – standard error.

**Table S5b: Genetic (upper matrix) and environmental correlations (lower matrix) of whole CC DTI measures and total WMLs for scan 2**

|  | **FA (ρ_g_ ± SE) [p]** | **MD (ρ_g_ ± SE) [p]** | **RD (ρ_g_ ± SE) [p]** | **AD (ρ_g_ ± SE) [p]** | **WMLs (ρ_g_ ± SE) [p]** |
| --- | --- | --- | --- | --- | --- |
| **FA (ρ_e_ ± SE) [p]** | 1 | -0.39 ± 0.13 [0.98 x 10^-2^]* | -0.73 ± 0.07 [1.86 x 10^-5^]* | -0.01 ± 0.16 [0.93] | -0.19 ± 0.12 [0.13] |
| **MD (ρ_e_ ± SE) [p]** | -0.50 ± 0.08 [3.61 x 10^-7^]* | 1 | 0.91 ± 0.02 [ 9.79 x 10^-7^]* | 0.88 ± 0.06 [ 1.22 x 10^-6^]* | 0.36 ± 0.13 [0.39 x 10^-2^]* |
| **RD (ρ_e_ ± SE) [p]** | -0.78 ± 0.04 [2.08 x 10^-24^]* | 0.92 ± 0.01 [3.63 x 10^-61^]* | 1 | 0.63 ± 0.11 [0.15 x 10^-3^]* | 0.34 ± 0.12 [0.58 x 10^-2^]* |
| **AD (ρ_e_ ± SE) [p]** | 0.26 ± 0.09 [0.78 x 10^-2^] | 0.53 ± 0.07 [7.41 x 10^-10^]* | 0.27 ± 0.09 [0.06 x 10^-1^] | 1 | 0.36 ± 0.13 [0.63 x 10^-2^]* |
| **WMLs (ρ_e_ ± SE) [p]** | -0.04 ± 0.10 [0.72] | -0.08 ± 0.11 [0.50] | -0.03 ± 0.11 [0.78] | 0.02 ± 0.10 [0.81] | 1 |

^*^Values are significant at p<0.01.

Notes: FA – Fractional anisotropy; MD- Mean diffusivity; RD- radial diffusivity; AD – Axial diffusivity; WMLs – White matter lesions; ρ_g_ – genetic correlation coefficient; ρ_e_ - environmental correlations; SE – standard error.

**Appendix- 1: Supplementary data:**

**Formulas used to calculate the DTI measures FA, MD, RD and AD**

The formulas for these DTI measures are based on the three Eigen values (ʎ_1_, ʎ_2_ and ʎ_3_) measured at each voxel. These Eigen values are quantified from the magnitude and direction of maximum and minimum water diffusion at each voxel. [1-3]

1. **FA (Fractional anisotropy)**

| FA= √½ √ [(ʎ_1-_ ʎ_2_)^2^ + (ʎ_1_ - ʎ_3_)^2^ + (ʎ_2_ - ʎ_3_)^2^] / √ (ʎ_1_^2^ + ʎ_2_^2^ _+_ ʎ_3_^2^) |
| --- |

1. **MD (Mean diffusivity)**

| MD = (*λ*_1_+ *λ_2_+* *λ_3_* /3) |
| --- |

1. **RD (Radial diffusivity)**

| RD *= λ* _┴_ *= (λ_2_+* *λ_3_* /*2*) |
| --- |

1. **AD (Axial diffusivity)**

| AD= *λ* _II_ = *λ*_1_ |
| --- |

**References:**

1. Basser PJ, Mattiello J and LeBihan D (1994) MR diffusion tensor spectroscopy and imaging. Biophysical Journal 66: 259-267.
2. Basser PJ and Pierpaoli C (1996) Microstructural and physiological features of tissues elucidated by quantitative-diffusion-tensor MRI. J Magn Reson B 111: 209-219.
3. Beaulieu C (2002) The basis of anisotropic water diffusion in the nervous system - a technical review. NMR in Biomedicine 15: 435-455.
